# Multiple Cold-Water Immersions Attenuate Muscle Damage but not Alter Systemic Inflammation and Muscle Function Recovery: A Parallel Randomized Controlled Trial

**DOI:** 10.1038/s41598-018-28942-5

**Published:** 2018-07-19

**Authors:** Angelina Freitas Siqueira, Amilton Vieira, Martim Bottaro, João Batista Ferreira-Júnior, Otávio de Toledo Nóbrega, Vinícius Carolino de Souza, Rita de Cássia Marqueti, Nicolas Babault, João Luiz Quagliotti Durigan

**Affiliations:** 1 Physical Education Program, University of Brasilia, Brasília, Brazil; 2 Physical Education Department, UDF University Center, Brasília, Brazil; 3 Physical Education College, University of Brasilia, Brasília, Brazil; 4 Federal Institute of Sudeste of Minas Gerais, Rio Pomba, Brazil; 5 Health Sciences Graduation Program, University of Brasilia, Brasília, Brazil; 6 Rehabilitation Sciences Graduation Program, University of Brasilia, Brasília, Brazil; 7 INSERM U1093, Faculty of Sport Science, University of Burgundy-Franche-Comté, Dijon, France

## Abstract

The aim of this study was to investigate the effects of multiple cold-water immersions (CWIs) on muscle function, markers of muscle damage, systemic inflammation and ECM degradation following exercise-induced muscle damage (EIMD). Thirty physically active males were randomly assigned to either a control (n = 15) or cold-water immersion (CWI) group (n = 15). The CWI group performed one immersion (10 °C for 20 min) at post-exercise and every 24 h for the following 72 h, while the control group remained in a seated position during these corresponding periods. Muscle strength, vertical jump height, muscle thickness, delayed-onset muscle soreness (DOMS), systemic creatine kinase (CK), C-reactive protein (CRP), inflammatory cytokines and matrix metalloproteinase-2 (MMP-2) activity were assessed at Pre, Post, 24, 48, 72, 96 and 168 h following EIMD. No significant time × group interaction was obtained for muscle strength, vertical jump height recovery and MMP-2 activity (p > 0.05). At 24 h, muscle thickness from the CWI group returned to baseline and was lower than the control (p = 0.04). DOMS returned to baseline at 168 h for the CWI group (p = 0.109) but not for the control (p = 0.008). At 168 h, CK showed a time-group difference with a greater peak for the control group (p = 0.016). In conclusion, multiple CWIs attenuated muscle damage, but not altered systemic inflammation and muscle function recovery.

## Introduction

The long-lasting impairment in muscle performance subsequent to unaccustomed and/or eccentric exercises has been referred as exercise-induced muscle damage (EIMD)^1^. EIMD causes an increase in inflammatory markers in the blood, associated with an increase of edema and delayed onset muscle soreness (DOMS), as well as a prolonged impairment in muscle strength, and range of motion^2^. Therefore, different strategies of muscle recovery have been employed to minimize EIMD. One strategy widely used today in both clinical settings and sports activities is cryotherapy using cold-water immersion (CWI)^3^ at temperatures lower than 15 °C^4^.

Although the mechanisms related to the benefits of CWI are not completely understood, it has been suggested that the decrease in muscle temperature causes a reduction in the metabolic rate, reactive oxygen species (ROS) production, and the inflammatory process, which may minimize secondary muscle damage^5^. In addition, alterations in intramuscular blood and lymphatic flow may occur partly because of cold-induced vasoconstriction and/or hydrostatic pressure^4^. It has been reported that the magnitude of the potential physiological changes related to CWI depends on the water temperature^6–8^, immersion duration, and frequency^3,9^. However, despite the widespread use of CWI, the optimal protocol to elicit the required physiological response remains unknown^3,9,10^.

The use of cryotherapy has been recommended for the first 72 h after muscle damage^11^. The theoretical hypothesis that underpins this recommendation is related to its intensive treatment during the destruction phase of muscle regeneration^12^. The destruction phase, lasting 0–72 h, is characterized by cell membrane rupture, myofiber death, muscle edema and the inflammatory process^13^. To our knowledge, few studies^14–17^ have explored the effects of multiple CWIs during the first 72 h after a single bout of EIMD on acute recovery and muscle function. Some of these studies demonstrated that this recovery strategy reduces subjective ratings of DOMS^16,17^ and perceived exertion^17^, attenuates indirect markers of muscle damage^14,16^, and accelerates the recovery of functional muscle performance^16,17^. However, there is conflicting evidence from previous studies and there is no clear consensus supporting the use of multiple CWIs administration in humans.

We have previously demonstrated in an animal model that multiple administrations of cryotherapy during the destruction phase can reduce the inflammatory process through a decrease in macrophage infiltration^18^. In the same study, cryotherapy also reduced the release of messenger RNA levels of tumor necrosis factor alpha (TNF-α), and extracellular matrix (ECM) degradation markers (matrix metalloproteinase-9, MMP-9)^18^. Siqueira and coauthors^19^ also reported that multiple cryotherapy administrations relieved the production of ROS after muscle injury. Since the balance of pro- versus anti-inflammatory cytokines plays a key role in muscle regeneration by affecting the activation of satellite cells and matrix metalloproteinases (MMPs), a clear understanding of the effects of multiple CWIs during the first 72 h following EIMD on the inflammatory process is important^20–23^.

Despite these promissory results and the widespread use of cryotherapy after muscle damage/injury in clinical practice, no study has addressed the effects of multiple CWIs on inflammation and ECM debradation markers. In addition, the results related to muscle function recovery remain to be determined in humans. Thus, we aimed to investigate the effects of multiple CWIs during the first 72 h following a single bout of EIMD on muscle function (i.e., muscle strength, and vertical jump height), markers of muscle damage (muscle thickness, DOMS and creatine kinase), systemic inflammation (C-reactive protein, and cytokine kinetics), and ECM degradation. Our hypothesis was that multiple CWIs during the first 72 h post-exercise would attenuate muscle damage and the inflammatory response, which would support a quicker recovery of muscle function.

## Results

We assessed the eligibility of thirty-two physically active males between December 2013 and August 2014. We excluded two individuals that had a thigh skinfold greater than 20 mm. Thirty individuals participated voluntarily and were allocated into the (1) control or (2) CWI group. However, one participant from the CWI group was excluded 48 h after exercise due to the manifestation of rhabdomyolysis diagnosed by the medical staff. Physical characteristics of each experimental group are shown in Table 1; there were no significant differences between groups at baseline (p > 0.05). The analysis of primary and secondary outcomes included all twenty-nine participants that finished the study.

**Table 1:** Age, body mass, height, body mass index (BMI), thigh skinfold, baseline knee extensors peak torque and countermovement jump (CMJ) height from subjects of each experimental group.

Physical characteristic	Control group (n = 15)	CWI group (n = 14)	*p* value
Age (years)	19.9 ± 1.4	20.5 ± 1.4	0.284
Body mass (kg)	71.3 ± 9.4	71.3 ± 9.1	0.997
Height (cm)	175.7 ± 7.2	176.3 ± 5.0	0.791
BMI (kg/m²)	23.0 ± 2.0	22.4 ± 2.8	0.496
Thigh skinfold (mm)	14.3 ± 5.8	13.6 ± 4.3	0.748
Peak torque (N·m)	333.5 ± 52.0	327.9 ± 47.1	0.770
CMJ height (cm)	46.6 ± 5.1	49.6 ± 8.1	0.297

Data are expressed as mean ± SD. The differences between groups were analyzed using Student’s test. No difference was observed in the physical characteristics between groups (p > 0.05). CWI: cold-water immersion.

### Muscle function

#### Muscle Strength

**Figure 1:**
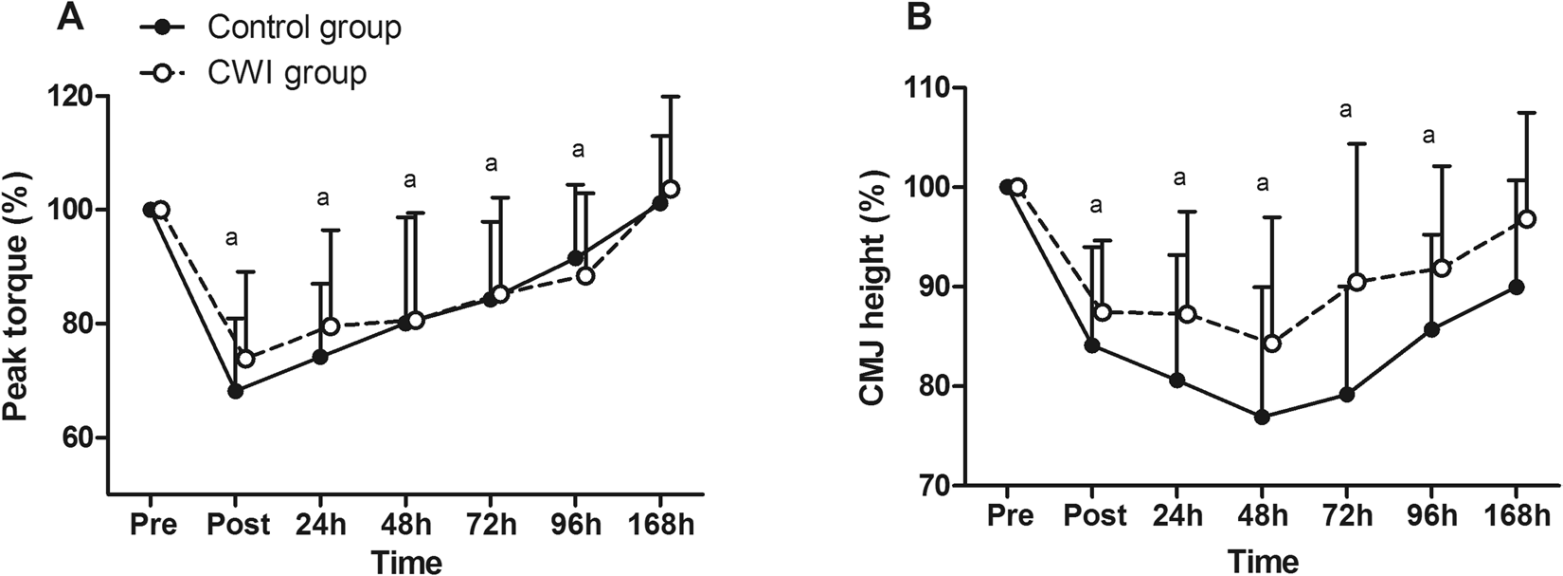
Changes in muscle function following exercise-induced muscle damage. (**A**) Knee extensor peak torque; and (**B**) counter-movement jump (CMJ) height. Data are expressed as mean ± SD. The differences within and between groups were analyzed by the two-way mixed-model ANOVA. ^a^Difference when compared to pre-value whatever the group. CWI: cold-water immersion.

No significant time × group interaction was obtained for muscle strength [F = 0.778, p = 0.588, η_ρ_^2^ = 0.027, power = 0.303]. Nevertheless, a significant main time effect was observed [F = 44.305, p < 0.001, η_ρ_^2^ = 0.613, power = 0.999]. The lowest values of muscle strength were obtained immediately post-exercise (Fig. 1A). Muscle strength returned to pre-values only at 168 h in both groups (p = 1.0).

#### Vertical jump height

Vertical jump height showed no significant time × group interaction [F = 1.220, p = 0.301, η_ρ_^2^ = 0.058, power = 0.465]. However, a significant main time effect was obtained [F = 17.116, p < 0.001, η_ρ_^2^ = 0.461, power = 0.999]. Vertical jump height declined immediately post-exercise with the lowest values at 48 h (Fig. 1B). Finally, the vertical jump height returned to pre-values at 168 h in both groups (p = 0.188).

### Markers of muscle damage

#### Muscle thickness

**Figure 2:**
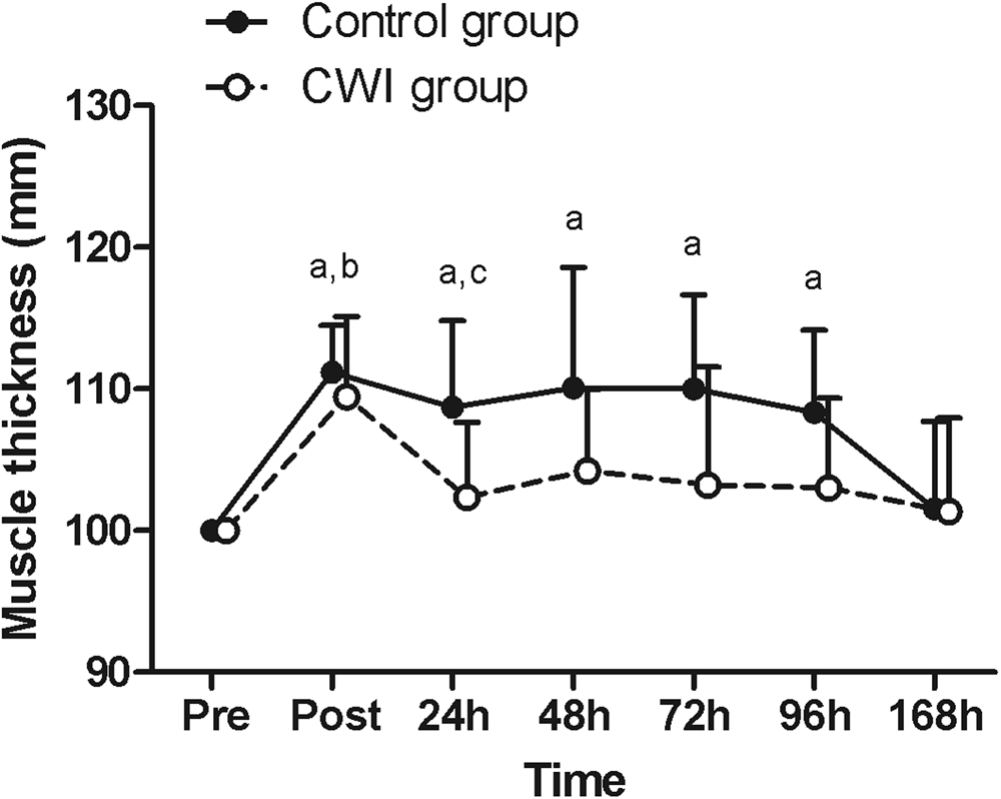
Changes in muscle thickness following exercise-induced muscle damage. Data are expressed as mean ± SD. The differences within and between groups were analyzed by the two-way mixed-model ANOVA and Tukey post-hoc test. ^a^Difference when compared to pre-value for the control group; ^b^difference when compared to pre-value for the CWI group; ^c^difference between control and CWI groups. CWI: cold-water immersion.

Muscle thickness showed a significant time × group interaction [F = 2.204, p = 0.04, η_ρ_^2^ = 0.109, power = 0.758] with a significant difference between groups at 24 h [F = 6.089, p = 0.024, η_ρ_^2^ = 0.253, power = 0.646]. A significant main time effect was also observed [F = 12.024, p < 0.001, η_ρ_^2^ = 0.400, power = 0.999]. The immediate increase in muscle thickness was similar in both groups, however, each group recovered differently over time (Fig. 2). Muscle thickness returned toward pre-values at 24 h in the CWI group (p = 0.900) and only at 168 h in the control group (p = 0.900).

#### Delayed-onset muscle soreness

**Figure 3:**
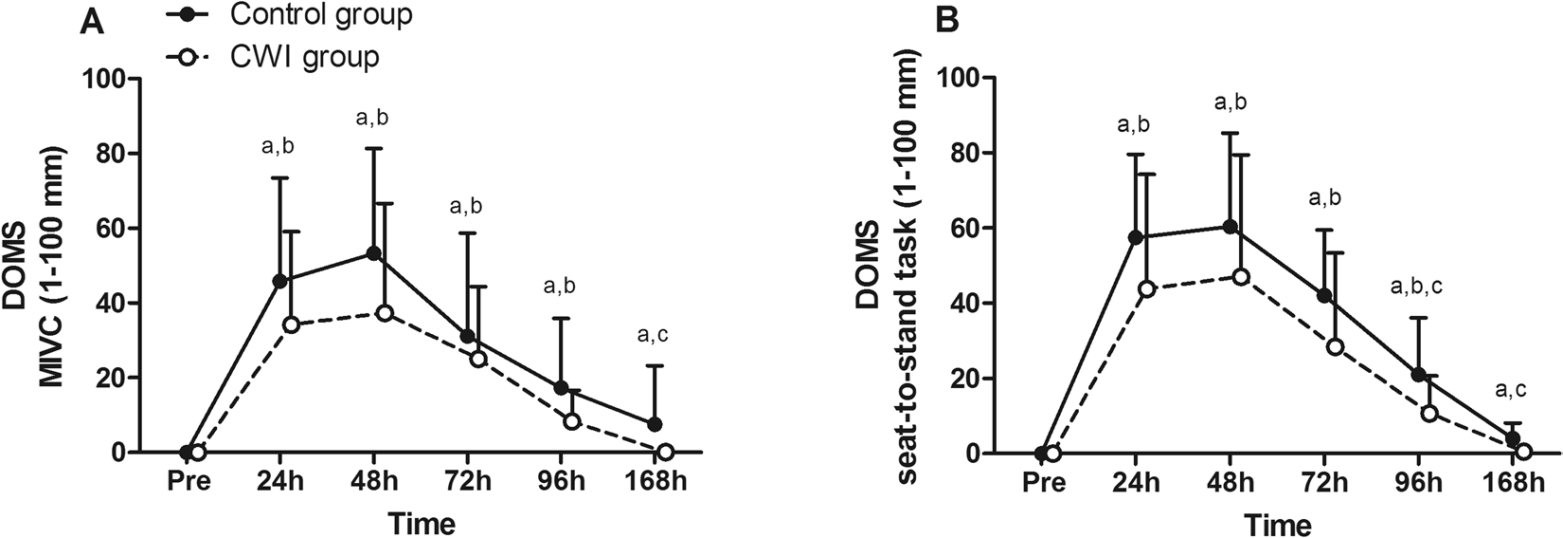
Changes in delayed-onset muscle soreness (DOMS) following exercise-induced muscle damage, during: (**A**) maximal voluntary isometric contraction (MIVC); (**B**) seat-to-stand task. Data are expressed as mean ± SD. The differences between groups were analyzed using a Mann-Whitney test and the differences in time for each recovery modality using the Wilcoxon test. ^a^Difference when compared to pre-value for the control group; ^b^difference when compared to pre-value for the CWI group; ^c^difference between control and CWI groups. CWI: cold-water immersion.

Time-group differences were found in both the maximum isometric voluntary contraction (MIVC) and the seat-to-stand assessment of DOMS: at 168 h (U = 30.5, p = 0.009, d = 0.678, power = 0.190) during MIVC (Fig. 3A); and at 96 h (U = 41, p = 0.046, d = 0.717, power = 0.468) and 168 h (U = 33, p = 0.014, d = 1.131, power = 0.626) during the seat-to-stand task (Fig. 3B). The CWI and control groups rated peak DOMS at 48 h post-exercise during both indices. The CWI group returned to pre-exercise values at 168 h (W = 1, p = 0.317, d = 0.318, power = 0.586 in MIVC; and W = 6, p = 0.109, d = 0.339, power = 0.368 in seat-to-stand task), whereas the control group did not recover over the investigated time period (W = 45, p = 0.008, d = 0.485, power = 0.163 in MIVC; and W = 45, p = 0.008, d = 1.017, power = 0.143 in seat-to-stand task).

### Biochemical markers of muscle damage, systemic inflammation and ECM degradation

There was no difference in the pre-values of any investigated biochemical markers between both experimental groups (p > 0.05, Table 2).

**Table 2:** Changes in biochemical markers following exercise-induced muscle damage.

Biochemical markers	Group	Time				
		Pre	24 h	48 h	72 h	168 h
CK (U/L)	Control	84.1 ± 31.3	313.5 ± 169.4^a^	231.8 ± 128.0^a^	840.4 ± 1707.1^a^	2932.0 ± 6446.9^a,c^
	CWI	86.4 ± 35.8	630.5 ± 1058.6^b^	216.3 ± 117.5^b^	127.5 ± 65.5	113.2 ± 61.1
CRP (mg/L)	Control	0.4 ± 0.4	0.8 ± 1.0	0.5 ± 1.1	0.3 ± 0.6	0.5 ± 0.8
	CWI	0.4 ± 0.5	1.2 ± 1.3^b^	0.5 ± 0.5	0.2 ± 0.2	0.4 ± 0.7
IL-6 (pg/mL)	Control	4.0 ± 4.9	5.8 ± 11.6	4.3 ± 8.9	1.0 ± 0.1	1.1 ± 0.4
	CWI	4.1 ± 10.4	5.9 ± 16.2	3.7 ± 8.8	10.9 ± 22.0	3.5 ± 6.9
TNF-α (pg/mL)	Control	20.3 ± 35.0	43.4 ± 60.2^a^	52.2 ± 106.4	13.8 ± 24.4	23.9 ± 31.6
	CWI	9.3 ± 19.1	30.3 ± 54.4	32.9 ± 334.6	7.8 ± 16.0	7.6 ± 13.9
IL-1α (pg/mL)	Control	25.4 ± 84.1	64.7 ± 147.5	36.6 ± 68.1	86.6 ± 142.7	100.7 ± 187.8
	CWI	23.4 ± 46.0	31.6 ± 68.1	31.5 ± 73.6	75.8 ± 120.3^b^	44.8 ± 91.0
IL-10 (pg/mL)	Control	129.4 ± 229.3	229.2 ± 555.9	505.7 ± 811.7	136.9 ± 252.9	164.1 ± 238.9
	CWI	39.5 ± 99.7	98.9 ± 178.3	85.5 ± 249.5	436.4 ± 1203.7	62.4 ± 145.6

Data are expressed as mean ± SD. The differences between groups were analyzed by a Mann-Whitney test and the differences in time for each recovery modality by Wilcoxon test. ^a^Difference when compared to pre-value of the control group; ^b^difference when compared to pre-value of the CWI group; ^c^difference between control and CWI groups. CK: creatine kinase; CRP: C-reactive protein; IL-6: interleukin-6; TNF-α: tumor necrosis factor alpha; IL-1α: interleukin-1 alpha; IL-10: interleukin-10; CWI: cold-water immersion.

#### Creatine kinase

Both experimental groups showed significant increases in CK activity at 24 h. The CWI group returned to pre-values at 72 h (W = 34.5, p = 0.155, d = 0.500, power = 0.547), remaining stable for all subsequent measurements (Table 2). However, the control group did not return to pre-values at any investigated time period (W = 63, p = 0.008, d = 0.442, power = 0.091 at 168 h). There was a time-group difference in CK activity at 168 h (U = 18, p = 0.016, d = 0.618, power = 0.162), as the control group interestingly reached peak CK activity.

#### Markers of systemic inflammation

Changes in markers of systemic inflammation are presented in Table 2. There was a significant increase in CRP levels at 24 h post-exercise in the CWI group (W = 52, p = 0.013, d = 0.430, power = 0.267). In regards to IL-6 and IL-10 levels, there was no difference between groups at any time point (p > 0.05) and no time effect (p > 0.05) for both control and CWI groups. TNF-α levels significantly increased at 24 h post-exercise in the control group (W = 28, p = 0.018, d = 0.334, power = 0.104) and IL-1α levels at 72 h in the CWI group (W = 15, p = 0.043, d = 0.413, power = 0.304).

#### Active MMP-2

MMP-2 activity showed no significant time × group interaction [F = 1.139, p = 0.356, η_ρ_^2^ = 0.125, power = 0.316] and no main time effect following EIMD [F = 1.415, p = 0.251, η_ρ_^2^ = 0.150, power = 0.389] (Table 3).

**Table 3:** Changes in MMP-2 activity following exercise-induced muscle damage.

Peak area	Group	Time				
		Pre	24 h	48 h	72 h	168 h
Active MMP-2 (p.d.u.)	Control	19,449 ± 4,767	21,774 ± 5,021	17,614 ± 8,067	19,963 ± 3,809	22,188 ± 4,733
	CWI	16,753 ± 9,275	21,988 ± 8,257	19,511 ± 8,431	19,420 ± 5,392	17,340 ± 5,877

Data are expressed as mean ± SD. The differences within and between groups were analyzed using the two-way mixed-model ANOVA. MMP-2: matrix metalloproteinase-2; p.d.u.: procedure defined unit; CWI: cold-water immersion. No difference was observed in the MMP-2 activity between groups (p > 0.05).

### Skin temperature, thermal sensation and thermal comfort

Skin temperature was similar between the control and CWI groups prior to the recovery procedures (control: 32.0 ± 1.7 °C vs CWI: 32.5 ± 1.4 °C, U = 95, p = 0.910, d = 0.316, power = 0.944). Throughout the 20 min recovery period, no significant skin temperature change was observed in the control group (p > 0.05, for all time-points). On the other hand, the CWI group had a significant decrease in skin temperature after the first 5 min of immersion (13.1 ± 2.4 °C, W = 7, p = 0.004, d = 6.070, power = 1.000). Over the remaining period of immersion, the skin temperature gradually reduced (10 min: 15.1 ± 1.3 °C; 15 min: 12.2 ± 1.7 °C; 20 min: 12.0 ± 1.5 °C), keeping relatively constant until the end and showing no difference between the time-points during the cooling procedures (p > 0.05).

Prior to recovery procedures, the median rates of thermal sensation were not different between both experimental groups (U = 95, p = 0.683, d = 0.190, power = 0.733). Participants from both groups rated their thermal sensation as “slightly warm”. However, the rates of thermal sensation were different between groups after 5 min of the recovery procedures (U = 5, p < 0.001, d = 2.752, power = 0.914). After 5 min into the cooling procedures, participants from the CWI group reported a “cool” sensation (W = 0, p = 0.001, d = 1.850, power = 0.321). Then, from 5 min until the end of the cooling procedures, they reported a “slightly cool” sensation (p = 0.001, for all time-points). Those from the control group reported a “neutral” sensation at 10 min and until the end of the recovery period (p < 0.05).

In regard to thermal comfort, there was no difference between groups prior to the recovery procedures, as both groups rated it as “comfortable” (U = 98, p = 1.000, d = 0, power = 1.000). After 5 min into the recovery procedures, a difference in thermal comfort rates between groups was observed (U = 182, p < 0.001, d = 2.264, power = 0.962). Compared to the baseline, the CWI group felt “slightly uncomfortable” during the cooling procedures (p = 0.002, for all time-points) while the control group continued feeling “comfortable” during the entire recovery period (p > 0.05).

## Discussion

The current study was conducted to determine the effects of a multiple CWIs recovery strategy on muscle function, markers of muscle damage, systemic inflammation and ECM degradation. The initial hypothesis was confirmed partially. Multiple CWIs during the first 72 h attenuated muscle damage by an early reduction of muscle thickness, and a delayed reduction of DOMS and CK activity. However, the recovery of maximal muscle strength, vertical jump height, systemic cytokine kinetics and ECM degradation did not differ between control and CWI groups. Our findings may help sports medicine professionals to better understand the effects of multiple CWI administrations on recovery following muscle-damaging exercise.

The present study demonstrated that four CWI administrations were not effective to accelerate the recovery of muscle function. Previous studies investigating the effectiveness of multiple CWIs during the first 72 h following a single bout of EIMD have reported conflicting results^14–17^. Discrepancies between the findings may be attributed to the CWI protocol (i.e. water temperature and duration). In an attempt to investigate the effects of different water temperatures during a multiple-CWI protocol, Machado and coauthors^17^ found that four immersions with durations of 15 min at 14 °C was more effective when compared to 9 °C. Moreover, Vaile and coauthors^16^ found that four immersions with durations of 14 min at 15 °C improved muscle function recovery. Therefore, a less harsh CWI temperature might optimize muscle function recovery^7,17^. Secondly, in the present study, CWI was applied with a longer duration (20 min) in contrast to the above mentioned studies (14 min^16^ and 15 min^17^). Actually, Peiffer and coauthors^24^ demonstrated that the magnitude of change in tissue temperature was associated with a longer CWI duration. Furthermore, it has been reported that an excessive tissue cooling could exacerbate the inflammatory response, which in consequence could even impair the recovery following EIMD^6^.

As expected, an increase in muscle thickness was observed immediately post-exercise in both groups. This parameter provides a measure of muscle swelling as a consequence of muscular and connective tissue damage, increased vessel permeability, or the combination of these factors^2^. We observed different muscle thickness kinetics between groups with the control group returning toward pre-values at 168 h, whereas the CWI group returned at 24 h post-exercise. CWI may have potentially reduced lymphatic and capillary cell permeability through peripheral vasoconstriction induced by low temperatures^25^ and/or the effect of hydrostatic pressure^4^. A decrease in fluid diffusion might assist in the reduction of the pro-inflammatory cell infiltration and edema formation^5^. In addition, time-course differences between CWI and control groups could be associated to the capacity of cryotherapy to decrease cellular metabolism and reduce pro-inflammatory cytokines and ROS release, which may minimize secondary muscle damage and preserve myofibers and other local muscle structures^19^.

Systematic reviews and meta-analyses have suggested that, for management of DOMS, CWI is better than conditions involving rest or no intervention^3,8–10^. Accordingly, the present study showed lower rates of DOMS in the CWI group compared to the control group at 168 h. This prolonged effect of DOMS is in agreement with a previous study that administered the same damaging procedure, even though a single bout of CWI showed no significant effect on DOMS^7^. Although, there is an inconsistency between the time of peak serum CK activity and peak DOMS, the longer presence of DOMS can be partially explained by the magnitude of muscle damage^26^. Among studies that investigated multiple CWIs effects^15–17^, only one^17^ has shown beneficial results, particularly, on soreness ratings immediately post CWI and 40 min post-exercise. Short-term analgesia after cryotherapy has been associated with reductions in the neural conductance velocity of sensory and motors neurons, which limits pain and reflexive spasms, respectively^27^. Beyond the CWI-invoked physiological changes already mentioned, long term analgesia might be ascribed to alterations in intracellular-intravascular fluid shifts which favor nutrition and waste transportation^28^ as well as attenuation of muscle edema, which prevents nerve compression^12^. Moreover, lower levels of pro-inflammatory cytokines could reduce rates of DOMS, since nociceptor activity in muscle tissues might be mediated by IL-1β, IL-6, and TNF-α^29^.

In accordance with previous studies, we observed that multiple CWIs were efficient in reducing CK activity following EIMD^14,16^. However, the studies showed differences on the time-points of reduction in CK activity, which might be due to the fatiguing exercise, the training level of the participants, as well as the CWI protocol. For instance, Eston and Peters^14^ observed a reduction in CK activity at 48 h and 72 h following a bout of eccentric exercise on the elbow flexors of the dominant arm and seven immersions at 15 °C for 15 min. Vaile and coauthors^16^ found an earlier reduction in CK activity, at 24 h and 72 h following an eccentric bi-lateral leg press protocol and four immersions at 15 °C with for 14 min. On the other hand, the present study demonstrated a later effectiveness on reduction in CK activity following a drop jump protocol and four immersions at 10 °C for 20 min, once the CK activity reduced only at 168 h. Indeed, the upper limbs muscles are more susceptible to muscle damage then lower limbs^30^. Moreover, the untrained participants of the present study were potentially more susceptible to muscle damage than the trained participants^2^ from the Vaile and coauthors^16^ study. Interestingly, the control group of the present study demonstrated a biphasic pattern of CK activity with a peak at 24 h post-exercise and a greater second peak at 168 h. Previous studies^7,31^ that used a similar protocol to induce muscle damage also found this biphasic pattern. This may be related to CK being leaked from the interstitial fluid and conducted to the lymphatic system before reaching the blood stream^2^. In addition, the time-course of CK may be related to the large inter-subject variability of CK release and clearance which can be influenced by many factors such as ethnicity, genetic factors, and ability to generate energy^32^.

An adequate balance between pro-inflammatory (such as TNF-α, IL-1α) and anti-inflammatory (such as IL-10) cytokines is important for muscle and surrounding connective tissue regeneration^20^. Furthermore, the magnitude of the inflammatory response affects muscle function^23^. In general, the ambiguous findings of inflammatory cytokines in the present study do not suggest that our multiple CWIs protocol would be more effective in reducing inflammatory responses than passive recovery. The results presented here showed that the CWI group had a significant increase in CRP levels at 24 h. In general, CRP is associated with the inflammatory response, because it would attract macrophages to the damaged tissue, among other roles^33^. Our results disagree with a recent meta-analysis that found differences in CRP levels favoring cooling at 48 h after EIMD^9^. Nevertheless, we must stress the heterogeneity of cooling modalities among the studies included in this meta-analysis^9^. Furthermore, the authors highlighted that this result may not represent the true effect of cryotherapy, since the few studies which measured CRP levels had only small sample sizes, and in the presence of one more inflammatory markers, no statistical difference between groups were found^9^.

IL-6 has been termed an “inflammation-responsive” myokine, since IL-6 is responsive to the mechanical loading associated with exercise, suggesting that it exerts a pro-inflammatory effect^20^; and on the other hand, IL-6 inhibits TNF-α production and enhances IL-10 production, suggesting that it plays a part in the anti-inflammatory effect^20^. White and coauthors^6^ noticed a relationship between long time periods (30 min) of a single bout of CWI protocols and significant increases in IL-6 levels immediately post and 2 h post immersion. The authors hypothesized that a longer duration of a single bout of CWI could lead multiple cell types, not only muscle cells, to produce IL-6, resulting in a sustained highly inflammatory process within 2 h post-exercise^6^. Previous studies showed that systemic concentrations of IL-6 usually return to baseline within 24 h post-exercise^6,34,35^. According to our data and others^16^, it remains unclear whether multiple CWIs could exacerbate IL-6 concentrations, since blood samples were not collected within 24 h post-exercise and immediately after each cooling procedure either. Moreover, a study into multiple whole body cryotherapy *versus* passive recovery, analyzing inflammatory markers within 24 h post-exercise, indicated no differences in IL-6 and IL-10 levels but revealed a significant increase in CRP and IL-1 receptor alpha and suppression of IL-1β in favor of the cryotherapy group^36^.

At 24 h post-exercise, the control group showed an increase in TNF-α levels. In fact, TNF-α mediates macrophage activation (to M1 phenotype), which in turn produces potent pro-inflammatory mediators, which together could start a breakdown of the damaged muscle and induce local edema^37^. Interestingly, it is possible to observe that changes in some biochemical markers (TNF-α and CK levels) during the early inflammatory phase coupled with long lasting edema and muscle performance impairment in the control group. The IL-1α increase observed in the CWI group at 72 h corroborates the hypothesis that reductions in muscle temperature by cryotherapy could delay the inflammatory cascade^38^. Muscle damage can cause the cell membrane to rupture and allow calcium ions to invade damaged tissue and activate calpain. Calpain performs the catalysis of the IL-1α precursor, making this molecule active^39^. Therefore, multiple cryotherapy administrations might have delayed IL-1α activation, until it was observed to peak in the blood stream at 72 h in the CWI group. Takagi and coauthors^38^ described that this delay in the inflammatory cascade after cryotherapy might be associated with an excessive deposition of collagen in damaged tissue. However, as the MMP-2 activity assessed in the present study was not changed even by exercise, it was not possible to suggest that collagen deposition was altered by CWI during muscle regeneration. Likewise, Tayebjee and coauthors^40^ found no alteration in MMP-2 following treadmill exercise testing. However, it was observed, even without any intervention, that there were participants showing high levels of MMP-2, in contrast to others being undetectable^40^. The authors highlighted the importance to elucidate the categorical distribution of MMP-2 concentration which remains unclear to date^40^. Interestingly, a study using an animal model showed that only high-intensity exercise promoted an increase in MMP-2 expression and mainly in muscles composed predominantly of fast fibers^41^. Most likely, these incongruent findings of MMP-2 following exercise also indicate the differences between intramuscular *versus* systemic analyses, as ECM degradation and remodeling have been explored using both plasma and muscle sample analyses^40,41^.

All recovery strategies, including CWI, have been challenged by the placebo effect^42^. Due to the popularity of CWI for recovery, it can be assumed that most participant had heard about CWI’s purported advantages, leading to a confounding influence on the assessments^10^. It has been demonstrated that the placebo effect not only influences subjective measures, but also muscle performance^43^. Broatch and coauthors^34^ performed a study that supported, at least in part, the placebo effect on the beneficial effects attributed to CWI. The experimental procedure made participants to believe that a liquid added to the thermoneutral water immersion was advantageous to muscle recovery^34^. Surprisingly, the placebo strategy was as effective as CWI on muscle strength recovery and ratings of readiness for exercise, pain, and vigor^34^. Whether the placebo effect affected the data from studies on CWI is an important issue that remains to be elucidated. Moreover, as previously observed^44^, subjective feeling during and after recovery modalities is of paramount importance. Even if no effect on performance was obtained, CWI could be perceived as effective. This feeling could bolster individuals’ positive attitudes toward subsequent exercises.

It is important to highlight the limitations in the present study. Firstly, we only focused on the use of multiple CWIs in young and healthy individuals. Although, the majority of participants in studies on CWI for preventing and treating DOMS were untrained, trained athletes are more likely to use CWI regularly^3^. Therefore, additional comparative studies are required investigating this population. Secondly, in order to determine regeneration processes, additional measurements would have been interesting. For example, muscle biopsies^45^ and intramuscular temperature^46^ measurements would have provided greater insight of local changes. Nonetheless, multiple immersions could increase the risk of contamination of the wound due to the invasive procedures. Also, exploring neuromuscular function using evoked contractile properties, voluntary activation measurements, and motoneuronal excitability would have helped to clarify the real physiological effects of CWI rather than any placebo effect. Thirdly, the addition of a single CWI group would have been interesting to determine whether a beneficial or a harmful magnitude effect exists between single and multiple immersions, especially regarding systemic inflammation and ECM degradation. Finally, although MMP-9 activity was detected in the zymography gels, MMP-9 was not measured because of the interference by coagulation/fibrinolytic pathways that may have increased the MMP-9 content in serum^47^.

In summary, multiple CWIs were effective to attenuate indirect markers of muscle damage, such as DOMS, muscle thickness, and CK activity. However, this recovery strategy appears to be ineffective on systemic inflammation and ECM degradation markers and muscle function recovery. Thus, the use of multiple CWIs could be recommended as a strategy that may reduce muscle damage following exercises, but without the expectative to enhance recovery between training sessions or competitive events.

## Methods

### Study design

Thirty physically active males voluntarily participated in this parallel randomized controlled trial study and were allocated into one of the two parallel groups: (1) control group or (2) CWI group. The cross-over design was not applied to avoid the influence of the repeated bout effect (faster recovery of muscle function after a second bout of similar eccentric-type exercise) on the magnitude of muscle damage between conditions^31,48^. This study was conducted according to the Declaration of Helsinki and approval for the project was obtained from the local ethics committee (University of Brasília Research Ethics Committee, Brasília, Brazil, protocol number 243/13). The trial was retrospectively registered at the US National Institutes of Health (ClinicalTrials.gov, on 16/12/2014, protocol number NCT02341612).

### Study population

Study inclusion required participants to be physically active males, practicing mild to moderate intensity aerobic activities (e.g. running and cycling) and/or recreational sports (such as soccer), 2–3 times per week. Participants were excluded if they: (1) had participated in regular strength training or intensive plyometric exercise during the last 3 months; (2) answered “yes” to any Physical-Activity Readiness Questionnaire (PAR-Q) questions^49^; (3) had any inflammatory disease or had taken any anti-inflammatory medications during the last 4 weeks; (4) had history of adverse reactions to cold temperatures; (5) had a thigh skinfold greater than 20 mm, as the amount of adipose tissue affects intramuscular cooling^50^; (6) had knee extensor torque less than 185 Nm, in order to achieve paired values between participants. Participants were instructed to maintain their usual hydration regimen; not to consume stimulants (e.g. alcohol, caffeine, chocolate) and anti-inflammatory medications; and not to exercise during their participation in the experiment. To avoid circadian influences, participants were asked to visit the Strength Laboratory of Physical Education Faculty at the same time of day each day, between 1 and 4 pm. Once informed of the purpose, procedures, discomforts, risks, and benefits, each participant signed an informed consent form.

Sample size was calculated based on the knee extensor peak torque data (considered the study’s primary outcome) that was reported in a similar study^51^, using G*Power (version 3.1.9.2; Heinrich Heine University Düsseldorf, Germany). The following design specifications were taken into account: α = 0.05; (1-ß) = 0.8; effect size ƒ = 0.2; test family = F test, and statistical test = analysis of variance (ANOVA) repeated measures, within-between interaction, groups = 2 and measurements = 7. Sample size estimation indicated 20 participants (10 per group). However, we decided to include more participants in order to increase statistical power.

#### Randomization

Following the participants’ eligibility screening, the randomization scheme was generated using the website Randomization.com.

### Study interventions

**Figure 4:**
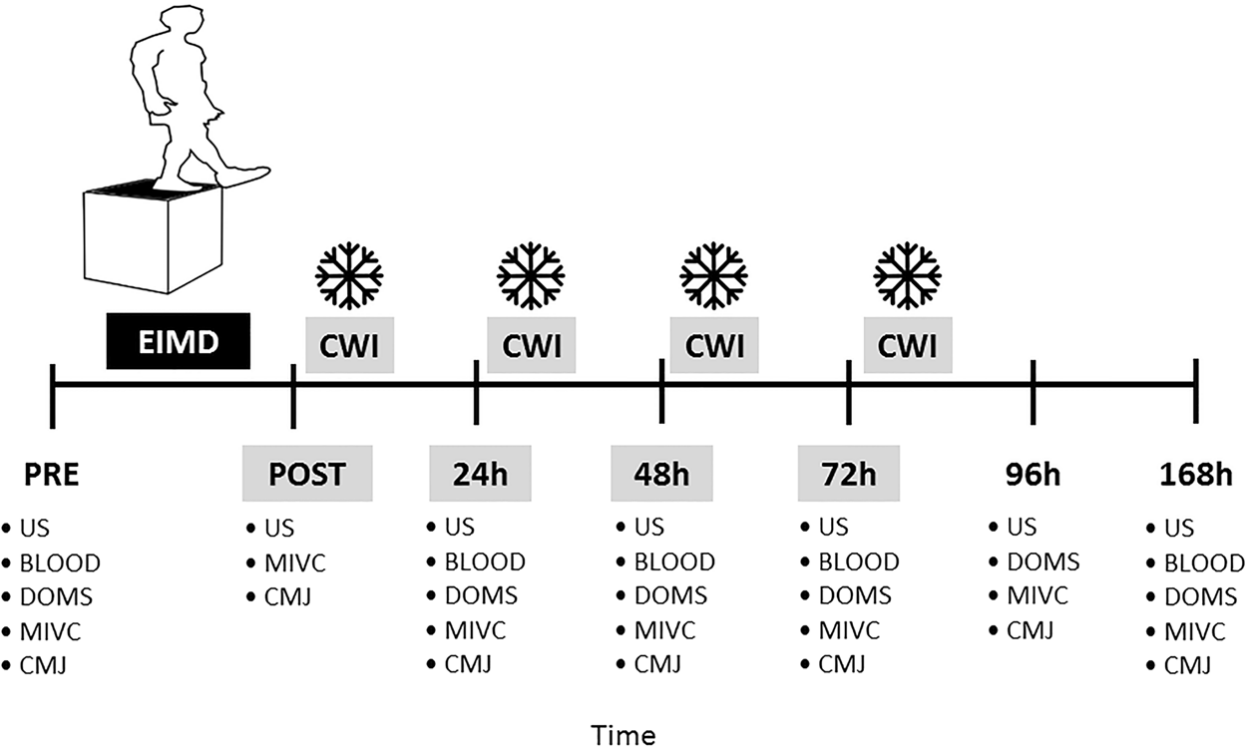
Experimental design. Represented are the times of each assessment procedure and multiple cold-water immersion (CWI) administrations. Maximum isometric voluntary contraction (MIVC), counter-movement jump (CMJ) and ultrasound (US) were measured at pre-exercise (Pre), immediately post (Post), 24 h, 48 h, 72 h, 96 h and 168 h post exercise-induced muscle damage (EIMD). Delayed onset muscle soreness (DOMS) was not measured at Post and blood samples (BLOOD) were not collected at Post and 96 h. Cold-water immersion (CWI) was performed 10 min post-exercise and every 24 h thereafter for the following 3 days after EIMD.

The participants visited the laboratory on seven occasions (Fig. 4). The first visit consisted of: (1) anthropometric measurements; and (2) familiarization with the experimental procedures. At three to seven days after familiarization, participants performed a muscle damaging protocol. In order to investigate the effects of multiple CWIs during the first 72 h following EIMD on muscle function, markers of muscle damage, systemic inflammation, and ECM degradation, were assessed in this sequence: (1) ultrasound assessment; (2) blood collection; (3) DOMS during seat-to-stand task; (4) peak torque and DOMS during MIVC; and (5) CMJ. These assessments were repeated: before exercise (Pre), and immediately (Post), 24, 48, 72, 96 and 168 h post-exercise. DOMS was not measured at Post, as it increases several hours’ post-exercise^26^. Blood samples were not collected at Post and 96 h, to limit the number of invasive measurements.

#### Protocol to induce muscle damage

The exercise protocol consisted of five sets of 20 drop jumps from a 60-cm box with two minutes of rest between sets^31^. After dropping down from the box and landing on the floor, participants were instructed to perform a maximal explosive vertical jump and then land on the floor. They were instructed to flex their knees to at least 90° (0° = full extension) during all landings and to keep their hands on their hips during the jumps. They were verbally encouraged to exert maximal effort during each jump repetition.

#### Cold-water immersion and passive recovery

CWI was applied 10 min post-exercise and every 24 h thereafter for the following three days. In total participants completed four bouts of immersion at 10 ± 1 °C for a duration of 20 min each. The duration was chosen based on studies that investigated a similar population^7,52^. Water temperature was selected based upon the most popular CWI range of 10 to 15 °C^3^. During the cooling procedure, participants remained seated with knees extended, while immersed up to the iliac crest, ensuring the lower limbs were fully submerged in the water bath. Water temperature was checked every 5 min and was maintained at the target temperature by adding crushed ice when necessary. Participants were instructed to make movements with their legs every 2 min to prevent the formation of the warmer boundary layer of water that forms immediately surrounding the skin. Participants in the control group remained comfortably seated at room temperature (21 ± 1 °C) during the corresponding periods of CWI sessions. After completing their respective interventions, subjects could continue with their regular daily activities.

### Study outcomes

#### Muscle function

**Muscle strength:** Maximal isometric voluntary knee extensor torque was measured at 60° (0° = full extension), using a commercial dynamometer (Biodex System 3, Biodex Medical, Inc., Shirley, New York, USA). Participants were comfortably positioned on the dynamometer seat with belts fastened across the trunk and pelvis to minimize body movements that could affect torque output^53^. The lateral epicondyle of the femur was aligned to the dynamometer’s axis and the chair and dynamometer settings for each subject were recorded during the familiarization session and were used throughout the study. Subjects were asked to cross their arms across the chest^54^ and to maximally contract their right knee extensors for 4 s^55^. They had two attempts to achieve their maximal isometric torque with 1 min of rest between attempts and received verbal encouragements throughout the tests^56^. Testing procedures were conducted by the same blinded examiner each time. The greatest torque was retained for further analysis.

**Vertical jump height:** CMJ height was measured using an AMTI force plate (model BP400600-HF-2000; Advanced Mechanical Technology, Inc., Watertown, MA, USA) with a sampling rate of 1,000 Hz. Subjects were asked to keep their hands on their hips and jump as high as possible. They had three attempts to achieve their best jump performance with 1 min of rest between attempts^7^. The greatest vertical displacement was considered as the maximal jump height, which was used for further analysis. A self-determined range of motion for the knee was permitted and they received verbal encouragement by the same blinded examiner. Data obtained during vertical jumps were captured from the manufacturer’s software (AMTI Acquisition Software, v 4.2; Advanced Mechanical Technology, Inc.) and processed using a custom MATLAB code (v R2008a7, The MathWorks Inc., Natick, MA, USA). From the ground reaction force, the force-displacement curve was calculated and then integrated to obtain the displacement of the center of mass at each instant of movement^57^.

#### Indirect markers of muscle damage

**Muscle thickness:** All sonograms were acquired by a portable ultrasound device (Philips-VMI, Ultra Vision Flip, Model BF, Minas Gerais, Brazil) equipped with a 7.5 MHz linear array transducer. A standardized protocol (including transducer placement, anatomic landmarks, and subject position) was set by the same examiner. It also included ultrasound settings (e.g. frequency, gain, tissue compression) that were kept constant between subjects and across all time periods, except the depth that was adjusted for each subject to display the entire muscle. Subjects were assessed in the supine position with their knee in maximal extension and neutral rotation. A water-soluble transmission gel was applied to the ultrasound probe to allow acoustic contact without depressing the dermal surface. The anterior images of the anterior thigh were obtained with the transducer placed perpendicular to the long axis of the thigh on its anterior surface, at 60% of the distance from the greater trochanter to the lateral epicondyle and 3 cm lateral to the midline of the anterior thigh^58^. Once the technician was satisfied with image quality, the image was kept for further analysis^59^. In order to assure replication of image location on repeated ultrasound assessments, a mark was drawn on the subject’s leg using indelible ink. All image measurements were performed by a blinded examiner in triplicate using Image J Software (NIH, Bethesda, MD, USA). Mean values were used as representative of the thigh muscles (rectus femoris and vastus intermedius) thickness. Muscle thickness, expressed in mm, was defined as the distance from the subcutaneous adipose tissue interface to the muscle-bone interface^60^.

**Delayed-onset muscle soreness:** Perceived muscle soreness of quadriceps muscles was assessed using a 100-mm visual analog scale. The scale ranged from “no soreness” (0) to “severe soreness” (100)^31^. Subjects rated their quadriceps soreness during two situations: (1) sit-to-stand task: three consecutive sit-to-stand movements from a 43-cm chair, which were performed with constant cadence (2 s to sit and 2 s to stand) and (2) MIVC^7^.

#### Biochemical markers of muscle damage, systemic inflammation and ECM degradation

**Blood samples and biochemical analyses:** Approximately 12 mL of blood was collected from the antecubital vein by the standard venipuncture technique using a commercially produced vacuum sealed kit. Tubes were centrifuged (Centrifugal machine, 3250 RPM, Model Centurion, São Paulo, Brazil) at room temperature for 20 min at 2500 rotations per minute (≈1000 × g). Serum was aliquoted (250 µL) and directly stored at −20 °C until analyzed by a blinded examiner. Blood analyses included biochemical markers of: muscle damage by measurements of creatine kinase (CK) activity; systemic inflammation by measurements of C-reactive protein (CRP) levels and cytokine levels, among them interleukin-6 (IL-6), tumor necrosis factor-alpha (TNF-α), interleukin-1 alpha (IL-1α) and interleukin-10 (IL-10); and ECM degradation by measurements of matrix metalloproteinase-2 (MMP-2) activity.

The biochemical analyses were done in duplicate, according to the manufacturers’ protocols by a blinded examiner. All calibration curves displayed linear coefficients (R2) ≥ 0.95 and inter-assay coefficients of variation <5%. CK activity was determined by enzymatic assay using a test kit for total CK (Siemens Medical System, Erlangen, Germany) with a limit of detection of zero (U/L) and linearity of the measurement of 1300 U/L. CRP levels were analyzed by latex particle enhanced immunoturbidimetric assay using a test kit for the width-range C-reactive protein (Siemens Medical Systems, Erlanger, Germany) with a limit of detection of 0.003 mg/L and linearity of the measurement of 156–164 mg/L. Serum levels of IL-6, TNF-α, IL-1α, IL-10 were obtained by commercial test kits from Quantikine® ELISA Human Immunoassay (R&D Systems, Inc., Minneapolis, USA) using an absorbance plate reader (ELx800, BioTek instruments, Inc., Winooski, EUA). Thresholds of detection were experimentally determined at 1.0 pg/mL for IL-6 and IL-10, 1.5 pg/mL for TNF-α and 0.4 pg/mL for IL-1α.

MMP-2 gelatinolytic activity was measured by zymography. Samples containing 0.5 µL of serum were added to 0.5 µL of SDS (8%) (v:v) and subsequently added 10 µL of buffer without β-mercaptoethanol-containing SDS (20%). Samples were resolved by electrophoresis in polyacrylamide gel containing SDS 10% (SDS_PAGE) and gelatin at a final concentration of 1 mg/mL. After electrophoresis, the gels were washed twice for 20 min in 2.5% of Triton X-100 to remove SDS. Gels were incubated in buffer substrate (50 mM Tris-HCl pH 8.0; 5 mM of CaCl^2^ and 0.02% NaN^3^) at 37 °C for 20 h. Gels were stained with Coomassie brilliant-blue for 1.5 h and distained with acetic acid: methanol: water (1:4:5) for activity bands visualization. The gelatinolytic activity was visualized as clear bands in the stained gel^61^. Densitometric semi-quantitative analysis of the MMPs protein bands was performed as previously described by Hu and Beeton^62^. The analyses were done in triplicate by a single blinded examiner using Image J Software (NIH, Bethesda, MD, USA) and the mean value of peak area were used in the final analysis.

**Thermal comfort**, **thermal sensation**, **and skin temperature:** Subjects were asked to rate their thermal sensation and thermal comfort before and every 5 min throughout the 20 min of each recovery procedure. Thermal sensation was rated on a nine-point Likert-type scale where “−4” = very cold, “−3” = cold, “−2” = cool, “−1” = slightly cool, “0” = neutral, “1” = slightly warm, “2” = warm, “3” = hot, and “4” = very hot. Thermal comfort was rated on a five-point Likert-type scale where “0” = comfortable, “1” = slightly uncomfortable, “2” = uncomfortable, “3” = very uncomfortable, and “4” = extremely uncomfortable^63^. Every 5 min, subjects were also asked to stand up and gently towel-dry their right thigh to have their skin temperature measured. The anterior thigh temperature was measured in the drawn landmark for ultrasound measurements (above the *rectus femoris* muscle). An infrared thermometer was used (Fluke, 566, China), which was kept perpendicularly positioned 8 cm from the skin. Over the 4 days, the mean value of skin temperature was calculated at each time-point in each group, as well as the median value of Likert-type scales.

### Statistical analysis

Data are described as means and standard deviation, except Likert-type measurement scales used for rates of thermal comfort and thermal sensation that are described as medians. The Shapiro-Wilk test was used to verify data distribution. Peak torque, CMJ height, muscle thickness and MMP-2 activity presented normal distributions. Thereby, they were analyzed using a two-way (group × time) mixed-model ANOVA. In case of significant main effect or interaction (time × group), a Tukey post-hoc test was applied. DOMS, CK, markers of systemic inflammation, skin temperature, and rates of thermal comfort and thermal sensation did not show a normal distribution and were analyzed using nonparametric tests. The Mann-Whitney test was performed to assess differences between groups and the Wilcoxon test was undertaken to evaluate differences in time for each recovery modality. The subjects’ physical characteristics, baseline peak torque and CMJ height were evaluated using an independent t-test. SPSS (Statistical Package for Social Sciences) version 20.0 (IBM, USA) was used for statistical analyses with an alpha level set at 5%. Additionally, effect sizes and statistical power were calculated. Effect sizes from data analyzed by ANOVA were determined using partial eta squared (η_ρ_^2^). Cohen^64^ has provided benchmarks to define small (η_ρ_^2^ = 0.01), medium (η_ρ_^2^ = 0.06) and large (η_ρ_^2^ = 0.14) effects^64^. To calculate the effect sizes and power from the data analyzed by non-parametric test, it was used the G*Power (version 3.1.9.2; Heinrich Heine University Düsseldorf, Germany). Effect sizes (d) from the data analyzed by non-parametric test were calculated with values of 0.2, 0.5 and above 0.8 considered to represent small, medium and large differences, respectively^64^. Power were calculated by the asymptotic relative efficiency (A.R.E.) method^65^.
